# Temporal Correlation Between Kawasaki Disease and Infectious Diseases in South Korea

**DOI:** 10.1001/jamanetworkopen.2021.47363

**Published:** 2022-02-07

**Authors:** Ji-Man Kang, Jaehun Jung, Young-Eun Kim, Kyungmin Huh, Jinwook Hong, Dong Wook Kim, Min Young Kim, Se Yong Jung, Jong-Hun Kim, Jong Gyun Ahn

**Affiliations:** 1 Department of Pediatrics, Severance Children’s Hospital, Institute for Immunology and Immunological Diseases, Yonsei University College of Medicine, Seoul, South Korea; 2 Artificial Intelligence and Big-Data Convergence Center, Gil Medical Center, Gachon University College of Medicine, Incheon, South Korea; 3 Department of Preventive Medicine, Gachon University College of Medicine, Incheon, South Korea; 4 Department of Big Data Strategy, National Health Insurance Service, Wonju, South Korea; 5 Division of Infectious Diseases, Department of Medicine, Samsung Medical Center, Sungkyunkwan University School of Medicine, Seoul, South Korea; 6 Department of Information and Statistics, Research Institute of Natural Science, Gyeongsang National University, South Korea; 7 Division of Pediatric Cardiology, Department of Pediatrics, Yonsei University College of Medicine, Seoul, South Korea; 8 Department of Social and Preventive Medicine, Sungkyunkwan University School of Medicine, Suwon, Gyeonggi-do, South Korea

## Abstract

**Importance:**

Infections are proposed to be triggering factors for Kawasaki disease (KD), although its etiological factors remain unknown. Recent reports have indicated a 4- to 6-week lag between SARS-CoV-2 infection and multisystem inflammatory syndrome in children with a similar presentation to that of KD.

**Objective:**

To investigate the temporal correlation between KD and viral infections, focusing on respiratory viruses.

**Design, Setting, and Participants:**

This cohort study was conducted among individuals aged 0 to 19 years diagnosed with KD between January 2010 and September 2020 from the Korean National Health Insurance Service. Data on infectious disease outbreaks from 2016 to 2019 were collected from the Korea Disease Control and Prevention Agency, Korean Influenza and Respiratory Virus Monitoring System, Korea Enteroviruses Surveillance System, and the Enteric Pathogens Active Surveillance Network in South Korea. Data were analyzed from December 2020 to October 2021.

**Main Outcomes and Measures:**

National databases for infectious diseases were used for a time-series analysis of the correlation between viral infections and KD. The temporal correlation between infectious disease outbreaks and KD outbreaks was evaluated using the Granger causality test (G-test), which is a useful tool to estimate correlations between 2 time series of diseases based on time lags.

**Results:**

Overall, 53 424 individuals with KD were identified, including 22 510 (42.1%) females and 30 914 (57.9%) males and 44 276 individuals (82.9%) younger than 5 years. Intravenous immunoglobulin-resistant KD was identified in 9042 individuals (16.9%), and coronary artery abnormalities were identified in 384 individuals (0.7%). Of 14 infectious diseases included in the analyses, rhinovirus infection outbreaks were identified as significantly correlated at 1 to 3 months before KD outbreaks in South Korea (*r* = 0.3; 1 month: *P* < .001; 2 months: *P* < .001; 3 months: *P* < .001). Outbreaks of respiratory syncytial virus infection were identified as significantly correlated with KD outbreaks by 2 months (*r* = 0.5; 2 months: *P* < .001). Additionally, varicella outbreaks were identified as significantly correlated at 2 and 3 months before KD outbreaks (*r* = 0.7; 2 months: *P* < .001; 3 months: *P* < .001).

**Conclusions and Relevance:**

In this cohort study with a time series analysis of children and youth in South Korea with KD, respiratory infections caused by rhinovirus and respiratory syncytial virus and varicella outbreaks were significantly correlated with KD at 1 to 3 months before KD outbreaks.

## Key Points

**Question:**

Is there a temporal association between respiratory viral infections and Kawasaki disease?

**Findings:**

This cohort study including 53 424 individuals with Kawasaki disease using national databases of infectious disease outbreaks found that outbreaks of respiratory infections caused by rhinovirus and respiratory syncytial virus and varicella were significantly correlated at 1 to 3 months before Kawasaki disease outbreaks in South Korea.

**Meaning:**

These findings suggest that viral infections, especially those caused by respiratory viruses, may be associated with triggering Kawasaki disease; therefore, trends in infectious diseases and Kawasaki disease incidence should be constantly monitored.

## Teaser

This cohort study assesses the temporal correlations between Kawasaki disease and viral infections in South Korea.

## Introduction

Kawasaki disease (KD) is an acute febrile vasculitis of childhood complicated by coronary artery lesions.^1^ The etiological characteristics of KD remain unknown; however, a potential association between KD and infectious diseases has been previously postulated.^2,3,4^ In South Korea, KD is most prevalent during winter, with the second peak in incidence occurring in late spring-summer.^5^ In addition, an increasing incidence of KD has been observed in several other countries, such as Japan and Taiwan.^6^ These epidemiologic features support the theory that infectious agents may be potential triggers of KD in children who are susceptible.

COVID-19 is caused by SARS-CoV-2 and was first reported in December 2019. Since the COVID-19 pandemic began, many countries worldwide, including South Korea, have implemented strong infection control measures based on nonpharmaceutical interventions (NPIs) to suppress the spread of the virus.^7^ NPIs are measures that help individuals and communities slow the spread of an epidemic without requiring pharmaceutical treatments and are also called *community mitigation strategies*. NPIs include social distancing, mask-wearing, intensive contact tracing and isolation, and environmental cleaning. Since SARS-CoV-2 mainly spreads via droplet transmission and direct contact, the NPI policies implemented to suppress SARS-CoV-2 have also been associated with changes in rates of infectious and infection-related diseases that are prevalent in a community.^8,9^ We previously reported that NPIs were associated with a decrease in the incidence of other respiratory infections^8^ and KD in South Korea,^9^ suggesting that infections may trigger KD occurrence.

COVID-19 is typically mild in children. However, cases of multisystem inflammatory syndrome in children (MIS-C) with a clinical presentation resembling that of KD have been recently reported in several countries.^10,11,12^ Most studies report a lag of several weeks between SARS-CoV-2 infection and the clinical presentation of MIS-C.^10,11,12,13^ This could provide an important insight into the inflammatory process of KD, with a presentation similar to that of MIS-C and suggests that an infectious trigger with a time lag may contribute to the pathogenesis of KD. With this background, we conducted an explorative investigation of whether viral infections precede KD, focusing on prevalent respiratory viruses in the community that have been mentioned as one of major triggering factors for KD.

## Methods

This cohort study was conducted in accordance with the Helsinki Declaration of the World Medical Association.^14^ The institutional review board of the Gachon University College of Medicine approved the study. The need for informed consent was waived owing to the retrospective nature of the study. This study is reported following the Strengthening the Reporting of Observational Studies in Epidemiology (STROBE) reporting guideline.

### Data Source for KD

The National Health Insurance Service (NHIS) is a universal health reimbursement insurer that covers approximately 98% of the South Korean population (51 780 000 citizens in the 2019 census) and has data on medical institutions, age, sex, diagnosis, prescriptions, and procedures covered by the NHIS. We selected the NHIS database as a data source for KD. KD was defined based on the *International Statistical Classification of Diseases and Related Health Problems, Tenth Revision* (*ICD-10*) diagnostic code (M30.3) of KD. In addition, the prescription code (Anatomical Therapeutic Chemical [ATC] code J06BA02) of intravenous immunoglobulin (IVIG) was also used to classify KD. IVIG-resistant KD was defined as KD with recrudescent or persistent fever 24 to 48 hours after the first IVIG infusion and was specified as IVIG-resistant KD when an IVIG prescription was required nonconsecutively for at least 3 days. KD complicated by coronary artery anomalies (CAAs) were identified with the diagnostic codes of CAAs (*ICD-10* codes, I25.4 and Q24.5). The prescription histories of the following medications were also collected: corticosteroids (ATC codes H02AB04, H02AB06, H02AB02, H02BX, HB02AB01, HB02AB07, HB02AB08, HB02AB09, HB02AB13, and HB02BX01), infliximab (ATC code L04AB02), and methotrexate (ATC codes L04AX03 and L01BA01). Lastly, the term *outbreak*, as used in this study, refers to an increased occurrence of a disease compared with what is expected in a community during a particular period.

### Data Source for Infectious Diseases

We used the national sentinel surveillance and notifiable infectious diseases databases operated by the Korea Disease Control and Prevention Agency. In the sentinel surveillance database, pathogens were identified through polymerase chain reaction (PCR) testing of appropriate specimens, which were collected weekly from participating institutions. We selected viral diseases with more than 100 cases reported annually during 2016 to 2019 in the Korean Influenza and Respiratory Virus Monitoring System (KINRESS), Korea Enteroviruses Surveillance System (KESS), and Enteric Pathogens Active Surveillance Network (EnterNet). Data from the KINRESS were collected for patients hospitalized with an acute respiratory illness, whereas data from the KESS and EnterNet were collected for outpatients who were symptomatic and hospitalized patients. Physicians are obliged by law to report any identified case of notifiable diseases. In the notifiable infectious disease database, we selected infectious diseases with more than 10 000 cases reported annually during 2016 to 2019 regardless of viral infection. Sexually transmitted diseases in the surveillance system and active tuberculosis among the notifiable diseases were excluded. Sexually transmitted diseases are generally not considered as a prevalent community infection in children, and acute tuberculosis tends to have a long incubation period that spans several months and up to 2 years.

### Statistical Analyses

Pearson correlation analysis was used to evaluate the correlation between the monthly incidence of KD and infectious disease outbreaks. The temporal correlation between infectious disease outbreaks and KD outbreaks was evaluated using the Granger causality test (G-test),^13^ which is a tool to estimate correlations between 2 time series of diseases based on time lags. Since the sentinel surveillance data is not population based, crude numbers of positive cases were used. All tests were 2-tailed, and *P* < .001 was considered statistically significant. Statistical analyses were performed using R software version 3.6.2 (R Project for Statistical Computing) and SAS software version 9.4 (SAS Institute). Data were analyzed from December 2020 to October 2021.

## Results

### Characteristics of Individuals With KD

From 2010 to 2020, 53 424 individuals with KD were identified, including 22 510 (42.1%) females and 30 914 (57.9%) males and 44 276 individuals (82.9%) younger than 5 years. IVIG-resistant KD was identified in 9042 individuals (16.9%), and KD with CAA complications was identified in 384 individuals (0.7%). The detailed characteristics of individuals with KD before and after the NPIs are presented in Table 1.

**Table 1.** Baseline Characteristics of Individuals With KD

Characteristics	Individuals, No. (%) (N = 53 424)
Sex	
Female	22 510 (42.1)
Male	30 914 (57.9)
Age, y	
<5	44 276 (82.9)
5 to <10	8647 (16.2)
10 to <20	501 (0.9)
Admission year	
2010	4934 (9.2)
2011	4853 (9.1)
2012	4941 (9.2)
2013	5469 (10.2)
2014	5679 (10.6)
2015	5475 (10.2)
2016	5255 (9.8)
2017	4837 (9.1)
2018	5173 (9.7)
2019	4690 (8.8)
2020	2118 (4.0)
IVIG-resistant KD^a^	9042 (16.9)
CAA complication	384 (0.7)
Treatment	
IVIG alone	48 480 (90.7)
Corticosteroids	4944 (9.3)
IVIG ≥3 d with corticosteroids	1859 (3.5)
Infliximab	76 (0.1)
Methotrexate	46 (0.1)

Abbreviations: CAA, coronary artery abnormality; IVIG, intravenous immunoglobulin; KD, Kawasaki disease.

^a^ IVIG-resistant KD was defined as recrudescent or persistent fever 24 to 48 hours after the first IVIG infusion.

### Monthly Distribution of Infectious Diseases

We included 11 viral diseases from the national sentinel surveillance database: respiratory syncytial virus (RSV), parainfluenza virus, adenovirus, coronavirus, metapneumovirus, rhinovirus, influenza virus, and bocavirus from the KINRESS; rotavirus and norovirus from EnterNet; and enterovirus from the KESS (Table 2). During 2016 to 2019, the annual mean (SD) reported incidence of respiratory viruses was 7322.5 (652.1) cases, including 2040 (224.4) rhinovirus cases, 1828 (335.1) influenza virus cases, 764.5 (220.8) adenovirus cases, 747.8 (37.9) parainfluenza virus cases, 576 (92.7) coronavirus cases, 576 (74.1) metapneumovirus cases, 547.5 (29.2) RSV cases, and 242.8 (75.4) bocavirus cases. The annual mean (SD) reported incidence for other viruses were 160.8 (72.8) rotavirus cases, 391.8 (75.9) norovirus cases, and 1704.3 (411.7) enterovirus cases (eTable 1 in the Supplement).

**Table 2.** Correlation Between the Monthly Incidence of KD and Infectious Disease Outbreaks^a^

Source	Pearson correlation		*P* value for time before KD out^a^		
	Correlation coefficient, *r*	*P* value	1 mo before KD	2 mo before KD	3 mo before KD
KINRESS					
Overall	0.7	<.001	.02	.01	.24
Respiratory syncytial virus	0.5	<.001	.09	<.001	.02
Parainfluenza virus	0.3	.02	.24	.47	.65
Adenovirus	0.5	<.001	.002	.01	.01
Coronavirus	0.5	<.001	.88	.01	.02
Metapneumovirus	0.2	.23	.08	.20	.35
Rhinovirus	0.3	.01	<.001	<.001	<.001
Influenza virus	0.4	.004	.15	.28	.37
Bocavirus	0.3	.01	.21	.26	.44
EnterNet					
Rotavirus	0.2	.17	.56	.53	.44
Norovirus	0.5	<.001	.56	.05	.09
KESS					
Enterovirus	0.1	.47	.79	.91	.48
Notifiable infectious diseases					
Varicella	0.7	<.001	.01	<.001	<.001
Mumps	0.3	.01	.02	.02	.03
Scarlet fever	0.4	.01	.08	.01	.02

Abbreviations: EnterNet, Enteric Pathogens Active Surveillance Network; KESS, Korea Enteroviruses Surveillance System; KINRESS, Korea Influenza and Respiratory Viruses Surveillance System; KD, Kawasaki disease.

^a^ The Granger causality test was used to estimate the correlation (precedence) between 2 time series of diseases using time lags.

Three notifiable infectious diseases (varicella, mumps, and scarlet fever) were included in the analyses. During 2016 to 2019, the median (range) annual reported incidences were 78 371 (54 060-96 467) cases of varicella, 17 291 (15 967-19 237) cases of mumps, and 14 522 (7562-22 838) cases of scarlet fever.

### Temporal Correlation Between KD and Infectious Diseases

**Figure 1.**
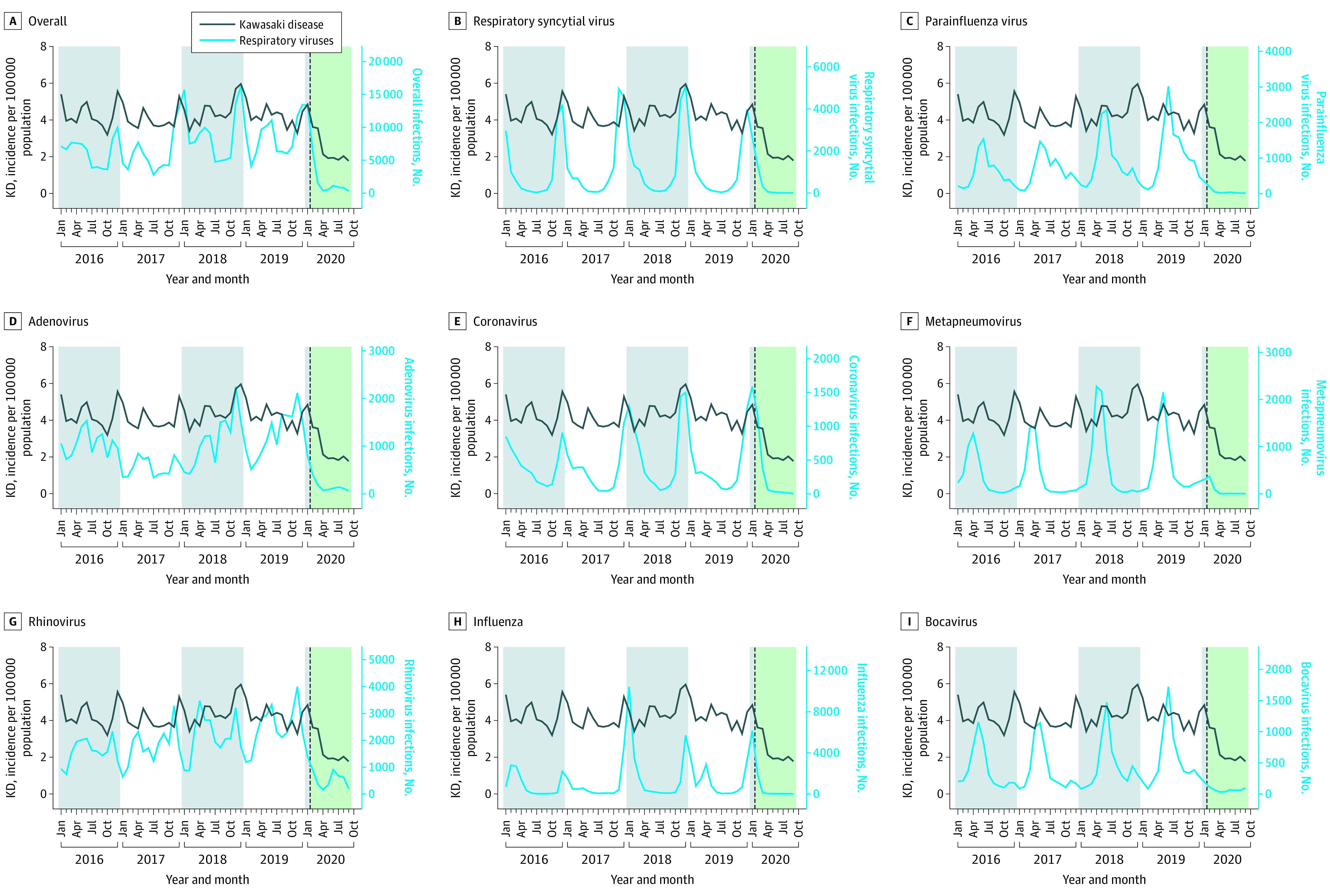
Monthly Respiratory Viral Infections From the Korea Influenza and Respiratory Viruses Surveillance System The dotted line represents February 2020, the time point at which nonpharmaceutical interventions for COVID-19 were implemented in South Korea. KD indicates Kawasaki disease.

**Figure 2.**
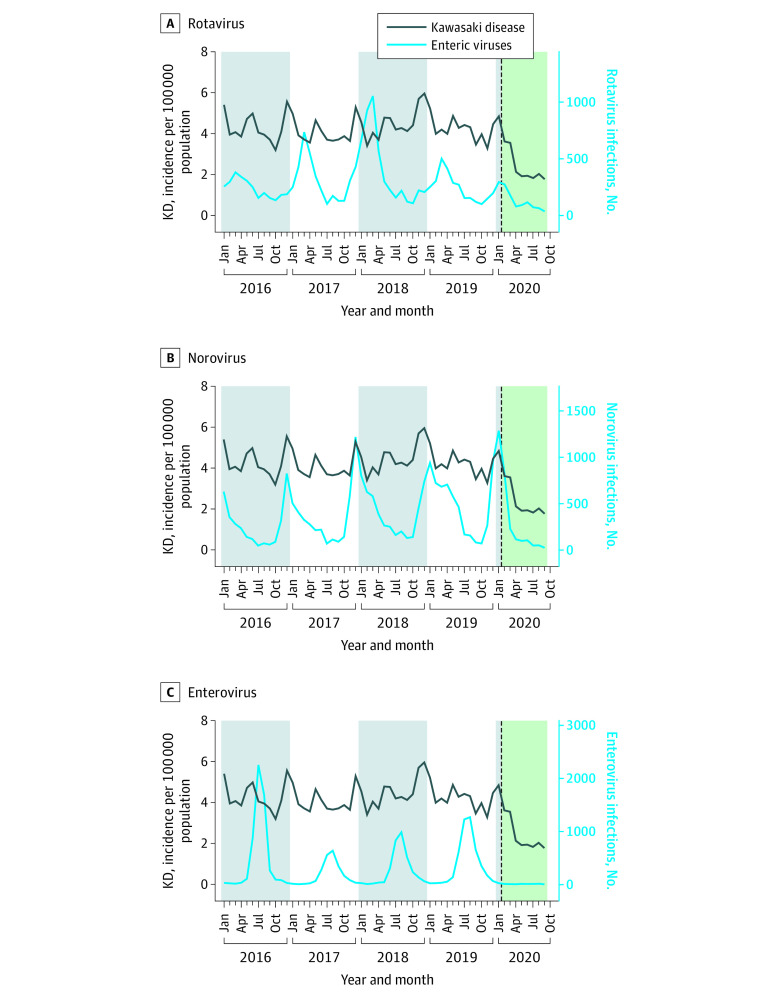
Monthly Viral Infections From the Korea Enteroviruses Surveillance System and Enteric Pathogens Active Surveillance Network The dotted line represents February 2020, when nonpharmaceutical interventions for COVID-19 were implemented in South Korea. KD indicates Kawasaki disease.

**Figure 3.**
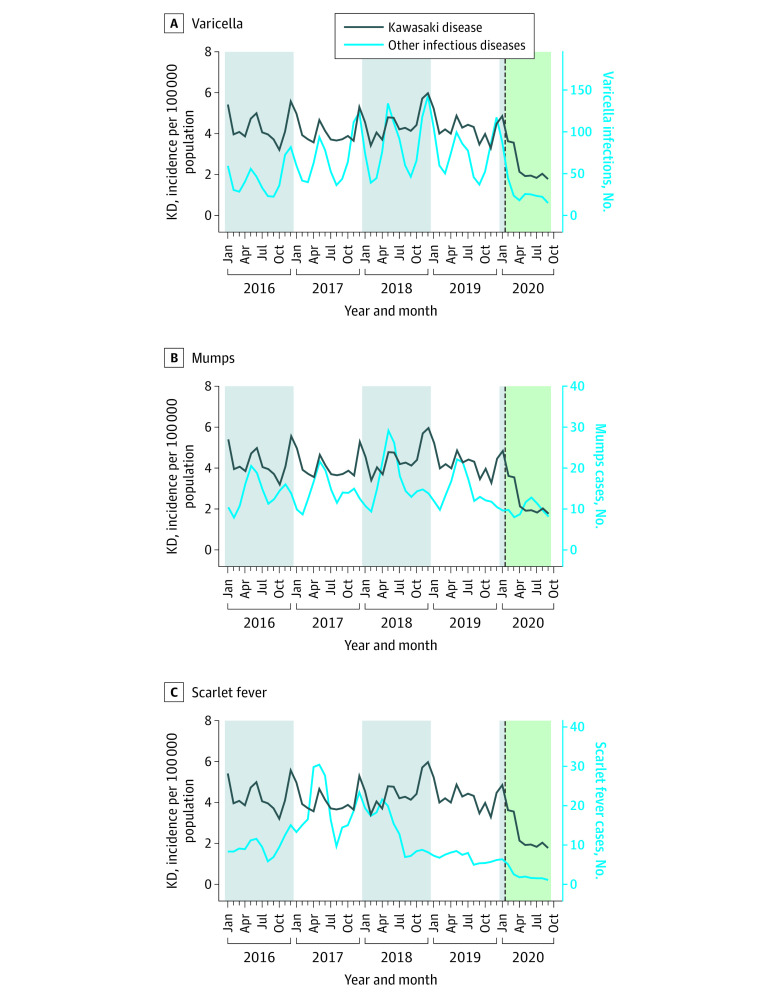
Monthly Incidence of Notifiable Infectious Diseases The dotted line represents February 2020, when nonpharmaceutical interventions for COVID-19 were implemented in South Korea. KD indicates Kawasaki disease.

Among respiratory viral infections, rhinovirus infection outbreaks were identified significantly correlated with KD outbreaks at 1 to 3 months (*r* = 0.3; 1 month: *P* < .001; 2 months: *P* < .001; 3 months: *P* < .001). RSV infection outbreaks were identified as significantly correlated with KD outbreaks at 2 months (*r* = 0.5; 1 month: *P* = .09; 2 months: *P* < .001; 3 months: *P* = .02) (Figure 1). Outbreaks of infections caused by other respiratory viruses (eg, enterovirus, norovirus, and rotavirus) were not correlated with KD outbreaks (Figure 2). Among the notifiable infectious diseases, varicella outbreaks were identified as significantly correlated at 2 and 3 months before KD outbreaks (*r* = 0.7; 1 month: *P* = .01; 2 months: *P* < .001; 3 months: *P* < .001). Outbreaks of mumps and scarlet fever, a bacterial infection caused by *Streptococcus pyogenes*, were not correlated with KD outbreaks (Figure 3). Additional results on the correlation between the monthly incidence of IVIG-resistant KD and infectious disease outbreaks are presented in eTable 2 in the Supplement.

## Discussion

This nationwide cohort study found that several viral infections, especially those caused by respiratory viruses, were outbreaks preceding KD outbreaks. A time-series analysis of KD and 14 infectious diseases found that varicella and respiratory infections caused by rhinovirus and RSV were significant precedent outbreaks that were correlated with KD development. In contrast, no temporal correlation was identified between KD outbreaks and outbreaks of infections caused by enteric viruses, such as norovirus, rotavirus, and enterovirus. Our findings highlight potential factors underpinning the etiological characteristics of KD.

Despite epidemiologic, clinical, and immunologic data suggesting that KD symptoms are associated with excessive immune responses triggered by an infectious agent in individuals with genetic susceptibility,^15^ the etiological characteristics of KD remain unclear. Observational epidemiologic evidence supports the theory that KD is correlated with infections, based on the commonalities between KD and other pediatric infectious conditions, such as the clinical similarities to infectious diseases in children, epidemiological temporal clusters, seasonal trends, and atypical recurrences.^4,16,17,18,19,20^ It has been suggested that viral disease is associated with KD based on virus detection in patients with KD,^21^ pathological based features,^22^ or temporal associations.^5^ In PCR-based analysis, concurrent respiratory viruses were detected in 42% to 50% of children with KD.^21,23^ A study by Chang et al^23^ reported that compared with a control group, patients with KD were more likely to have test results positive for various viruses, including enterovirus (16.8% vs 4.4%; *P* < .001), adenovirus (8.0% vs 1.8%; *P* = .007), human rhinovirus (26.5% vs 9.7%; *P* < .001), and coronavirus (7.1% vs 0.9%; *P* = .003), while a study by Turnier et al^21^ reported rhinovirus or enterovirus as the most commonly identified virus (28%) in children with KD, followed by adenovirus, RSV, and human metapneumovirus (5% each). Pathology research by Rowley et al^22^ found RNA-containing intracytoplasmic inclusion bodies in lung tissues from patients who died of late-stage KD, suggesting that KD may be caused by an undiscovered RNA virus that results in persistent infection in macrophages. Their findings support that intracytoplasmic inclusion bodies found in bronchial epithelial cells and a variety of different cell types across the body contain RNA and could be linked to the KD causative agent. However, the suspected KD-associated RNA virus has yet to be identified. In a temporal association study between the monthly occurrence of KD and detection of viruses,^5^ the monthly incidence of KD was correlated (*r* = 0.382; *P* = .02) with the monthly total virus detection. Human bocavirus and enterovirus, in particular, had significant correlations with monthly patterns of KD occurrence, while influenza virus did not have a significant correlation with KD occurrence. These findings support that viruses are associated with KD occurrence.

To our knowledge, no other study has analyzed the correlation between precedent viral infections with a time difference of 1 to 3 months and the occurrence of KD. Notably, outbreaks of several viral infections were observed to precede KD outbreaks in this study. Previous studies have suggested that infections caused by respiratory viruses, including adenovirus, rhinovirus, enterovirus, and coronavirus, are triggering factors for KD.^4,5,24,25^ However, owing to the seasonality of these viruses, distinguishing whether a concurrent infection is a triggering factor has been challenging.^24,25,26^ Regarding MIS-C, which shares clinical features with KD, recent studies have reported an increase in the number of MIS-C cases 4 to 6 weeks after peaks of COVID-19 cases.^10,11,12,13^ Thus, we investigated the prevalence of infectious diseases before KD outbreaks and observed that outbreaks of infections caused by rhinovirus and RSV and varicella were significantly correlated at 1 to 3 months before KD outbreaks. However, no such correlation was noted for infections caused by respiratory viruses, such as adenovirus, parainfluenza virus, coronavirus, metapneumovirus, influenza virus, and bocavirus, nor for enteric viruses, mumps, and scarlet fever. Our findings could provide clues regarding potential triggering factors that may explain KD pathophysiological pathways. Nevertheless, careful interpretation is warranted, given that this study only looked at correlation and not causation.

We previously reported that broad and intensive NPIs were associated with reduced incidence of respiratory infections.^8^ In addition, we observed a decrease in KD incidence by approximately 40% after the implementation of NPIs.^9^ Our current hypothesis is based on the simultaneous decrease in the incidence of respiratory infections and KD associated with the implementation of nationwide NPIs, alongside the evidence that infectious diseases may be predisposing factors for KD. Nevertheless, it is necessary to evaluate the associations of environmental factors other than reduced incidence of infections with KD incidence. Recent studies investigating the correlation between KD incidence and air pollutants have reported conflicting results.^27,28,29^ Lin et al^27^ and Zeft et al^28^ reported no association, whereas Jung et al^29^ reported a significant association between exposure to ozone and the risk of KD. In addition, mercury, dust mites, rug shampoo, and pollen release are hypothesized to contribute to KD pathogenesis, although conclusive evidence to support a direct causal effect between these environmental factors and KD is lacking.^30,31^ To date, the only consistent evidence on the associations of NPIs with environmental factors is a decrease in the incidence of respiratory infections.^8^ Furthermore, the large association of NPIs with KD incidence is likely due to an attenuation of environmental triggering factors, given that genetic susceptibility and immune system function are unlikely to have changed significantly on a population level over such a short timeframe. Collectively, our data suggest that respiratory infections may be triggering agents for KD.

This study has several strengths. First, to our knowledge, it is the first ecological study to use a time series analysis over 11 years to evaluate the incidence of KD and preceding viral infections at a national level. Second, we introduced the concept of a temporal correlation, which has also been observed between MIS-C and SARS-CoV-2 infections, to further clarify the link between viral infections and KD.

### Limitations

This study has some limitations. First, based on claims data policies of the NHIS, a small number of unclaimed KD cases may have been excluded. However, claim patterns are unlikely to have changed significantly during the study period. Second, other factors indirectly associated with NPIs, such as health care–seeking behaviors, may have resulted in bias. Third, the national sampling surveillance system, including the KINRESS, does not monitor the total number of cases of the infections included in our analysis and therefore may not fully reflect the incidence of community infections. Nevertheless, the KINRESS is a national surveillance system involving 196 medical institutions nationwide and provides a sufficient representation of domestic respiratory viral infection trends in South Korea.

## Conclusions

This time-series cohort study of KD and 14 infectious diseases found that respiratory infections caused by rhinovirus and RSV and varicella were significant outbreaks that occurred 1 to 3 months before KD outbreaks. In contrast, no temporal correlation was observed between KD outbreaks and outbreaks of infections caused by enteric viruses, such as norovirus, rotavirus, and enterovirus. Our findings suggest a temporal correlation between a set of respiratory viral infections preceding onset of KD in a large complete national data from South Korea. As such, constant monitoring of trends in infectious diseases and KD incidence is recommended. It would also be interesting to perform universal PCR viral testing in patients with acute KD to get a better profile of the viral agents present in that population. This, combined with temporal correlations, would help advance our knowledge of the etiological characteristics of KD.

## Notes

Supplement. **eTable 1.** Annual Incidence of Viral Infections Monitored by the National Sentinel Surveillance System in Korea **eTable 2.** Correlation Between the Monthly Incidence of IVIG-R and Infectious Disease Outbreaks
