# Supplementary material for: Temporal Correlation Between Kawasaki Disease and Infectious Diseases in South Korea
Source: JAMA Netw Open. 2022 Feb 7;5(2):e2147363. doi: 10.1001/jamanetworkopen.2021.47363 (PMC8822386; doi:10.1001/jamanetworkopen.2021.47363)
Supplement: Supplement. — eTable 1. Annual Incidence of Viral Infections Monitored by the National Sentinel Surveillance System in Korea eTable 2. Correlation Between the Monthly Incidence of IVIG-R and Infectious Disease Outbreaks [file jamanetwopen-e2147363-s001.pdf]

Supplemental Online Content

Kang JM, Jung J, Kim YE, et al. Temporal correlation between Kawasaki disease and infectious diseases in South Korea. *JAMA Netw Open*. 2022;5(2):e2147363. doi:10.1001/jamanetworkopen.2021.47363

eTable 1. Annual Incidence of Viral Infections Monitored by the National Sentinel Surveillance System in Korea
eTable 2. Correlation Between the Monthly Incidence of IVIG-R and Infectious Disease Outbreaks

This supplemental material has been provided by the authors to give readers additional information about their work.

© 2022 Kang JM et al. *JAMA Network Open.*

eTable 1. Annual Incidence of Viral Infections Monitored by the National Sentinel Surveillance System in Korea

	2016	2017	2018	2019	Annual cases, average (SD)	
Korean Influenza and Respiratory Virus Monitoring System						
Respiratory viruses	6682	67800	7529	8299	7322.5	(652.1)
Adenovirus	735	445	814	1064	764.5	(220.8)
Bocavirus	166	241	199	365	242.8	(75.4)
Coronavirus	638	530	687	449	576	(92.7)
Influenza	1743	1295	2032	2242	1828	(335.1)
Parainfluenzavirus	698	767	728	798	747.8	(37.9)
Respiratory syncytial virus	523	540	530	597	547.5	(29.2)
Rhinovirus	1727	2329	1953	2151	2040	(224.4)
Metapneumovirus	452	633	586	633	576	(74.1)
Enteric Pathogens Active Surveillance Network	527	769	417	497	552.5	(131.3)
Rotavirus	124	283	143	93	160.8	(72.8)
Norovirus	403	486	274	404	391.8	(75.9)
Korea Enteroviruses Surveillance System*						
Enterovirus	n/a	1312	2273	1528	1704.3	(411.7)

SD=standard deviation

*The Korea Enteroviruses Surveillance System has been implemented since 2017.

© 2022 Kang JM et al. *JAMA Network Open.*

eTable 2. Correlation Between the Monthly Incidence of IVIG-R and Infectious Disease Outbreaks*

	**Pearson correlation**		**Lag 1 (1 month before KD)***	**Lag 2 (2 month before KD)***	**Lag 3 (3 month before KD)***
	**Correlation coefficient (r)**	***P*-value**	***P*-value**	***P*-value**	***P*-value**
**Korea Influenza and Respiratory Viruses Surveillance System (KINRESS)**					
Overall	0.4	<.001	0.33	0.79	0.89
Respiratory syncytial virus	0.3	0.02	0.36	0.01	0.01
Parainfluenza virus	0.2	0.06	0.32	0.58	0.81
Adenovirus	0.5	<.001	0.01	0.01	0.01
Coronavirus	0.4	0.01	0.87	0.01	0.01
Metapneumovirus	0.1	0.37	0.41	0.58	0.57
Rhinovirus	0.4	0.01	<.001	<.0001	<.001
Influenza virus	0.3	0.02	0.27	0.44	0.63
Bocavirus	0.2	0.09	0.50	0.37	0.58
**Enteric Pathogens Active Surveillance Network (EnterNet)**					
Rotavirus	0.1	0.78	0.79	0.96	0.93
Norovirus	0.5	<.001	0.77	0.04	0.09
**Korea Enteroviruses Surveillance System (KESS)**					
Enterovirus	0.1	0.94	0.95	0.59	0.63
**Notifiable infectious diseases**					
Varicella (chickenpox)	0.5	<.001	0.09	0.01	<.001
Mumps	0.1	0.59	0.33	0.21	0.34
Scarlet fever	0.3	0.04	0.79	0.38	0.47

*The Granger causality test was used to estimate the correlation (precedence) between two time series of diseases using time lags. IVIG-R, intravenous immunoglobulin- resistant Kawasaki disease.

© 2022 Kang JM et al. *JAMA Network Open.*
